# More Than Meets the Eye: Idiopathic Orbital Inflammation Mimicking Orbital Cellulitis

**DOI:** 10.7759/cureus.12655

**Published:** 2021-01-12

**Authors:** Nicholas C Lee, Jaspreet Loyal, Adam Berkwitt

**Affiliations:** 1 Internal Medicine & Pediatrics, University of Texas Southwestern, Dallas, USA; 2 Pediatrics, Yale School of Medicine, New Haven, USA

**Keywords:** orbital myositis, idiopathic orbital inflammation, orbital cellulitis, preseptal cellulitis, pediatrics

## Abstract

An 18-year-old female presented with left eye periorbital swelling, erythema, and pain for three days. Computed tomographic images showed swelling of the medial rectus muscle, and she was diagnosed with orbital cellulitis and initiated on empiric antibiotics. Over the next 48 hours, she did not clinically improve, resulting in an MRI and further workup of infectious, oncologic, endocrinologic, and rheumatologic etiologies was unrevealing and ruled-out malignancy, sarcoidosis, Wegner’s, and thyroid eye disease.

Given the negative workup, the presentation was determined to be consistent with idiopathic orbital inflammation (orbital myositis variant) via a diagnosis of exclusion. Therefore, the patient was empirically treated with intravenous steroids that produced pronounced improvement within 24 hours. The patient was discharged in improved condition with a prednisone taper and rheumatology follow-up. Idiopathic orbital inflammation is a rare diagnosis of exclusion in pediatrics that merits prompt consideration and work-up if treatment for orbital cellulitis does not progress as expected.

## Introduction

Pediatricians are frequently confronted with orbital swelling as a chief concern, and the instinctive differential diagnosis usually centers on orbital versus pre-septal cellulitis. Our case report illustrates an 18-year-old girl who was admitted with presumed orbital cellulitis that required a broad differential of infectious, oncologic, endocrine, and rheumatologic etiologies. The value of this case lies in providing a broad approach to considerations of periorbital swelling beyond infection and the extensive process required to establish a rare diagnosis of exclusion. The incidence of idiopathic orbital inflammation (IOI) is unknown due to its rarity, yet it is estimated that only 6-17% of all IOI cases occur in the pediatric population [1]. The etiology remains unclear, but it may be related to an underlying rheumatologic or infectious process.

## Case presentation

An 18-year-old female with obesity and mild intermittent asthma presented to the pediatric emergency department after three days of progressive left eye swelling, erythema, and pain with lateral ocular movement. The pain was retro-orbital, quantified as 8/10, and worsened with left eye abduction. There was no improvement in pain with warm compresses or acetaminophen. She denied fever, headache, visual changes, eye discharge, rhinorrhea, nasal congestion, rash, fatigue, and/or weight loss. Both her family and social history were non-contributory. 

**Figure 1:**
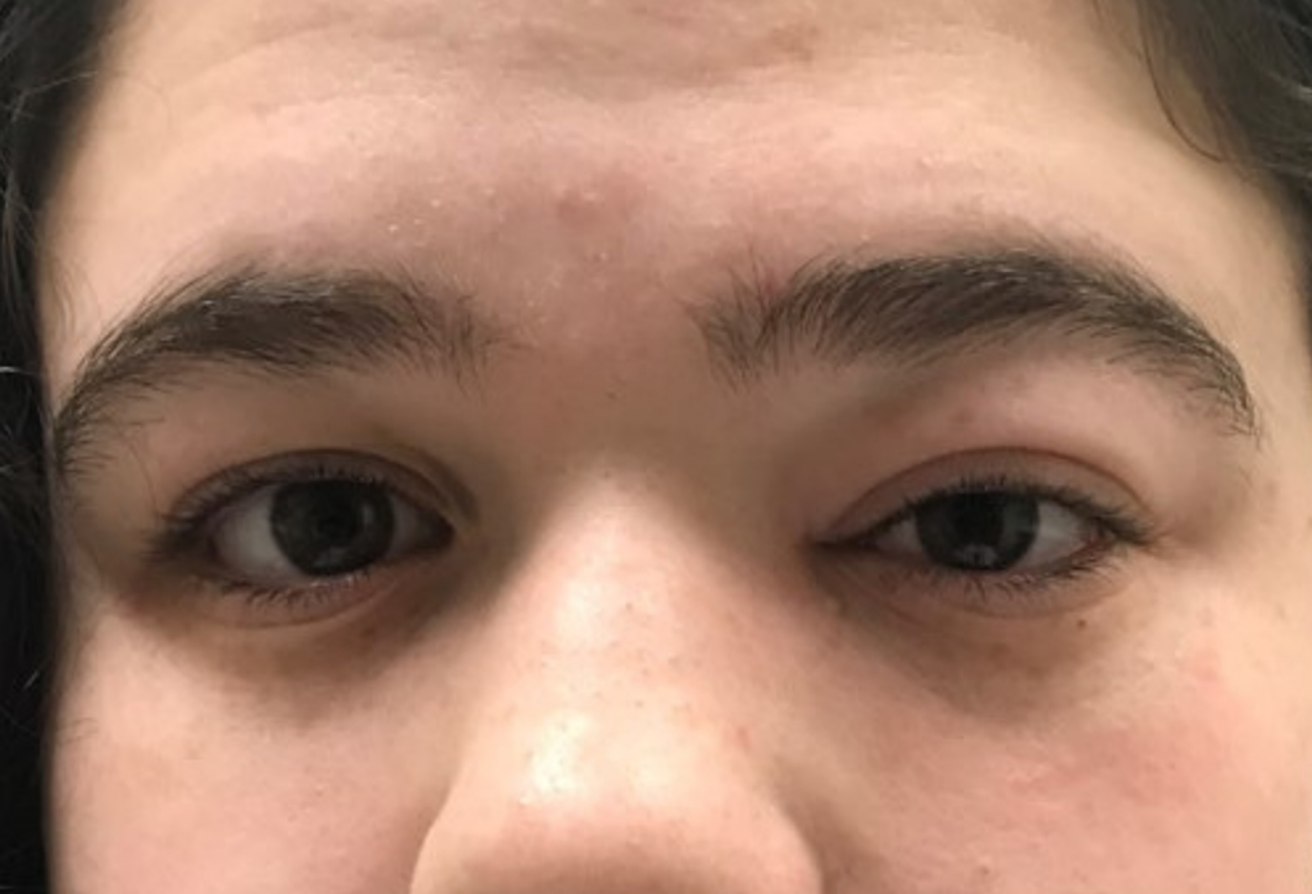
Eye examination on initial presentation

On physical exam, her temperature was 96.8^o^F and the remainder of her vital signs were normal for age. Initial ophthalmologic exam demonstrated moderate edema and erythema of the left upper and lower lids, mild proptosis, and pain on left lateral gaze (Figure 1). Intraocular pressures and funduscopic exam were normal. Her pupils were both equal and reactive to light and her extraocular movements were intact. She had no sinus tenderness, her oropharynx was clear, her neck supple without masses, and her pulmonary, cardiac, abdominal, neurologic, and dermatologic exams revealed no deficits and/or abnormalities.

**Figure 2:**
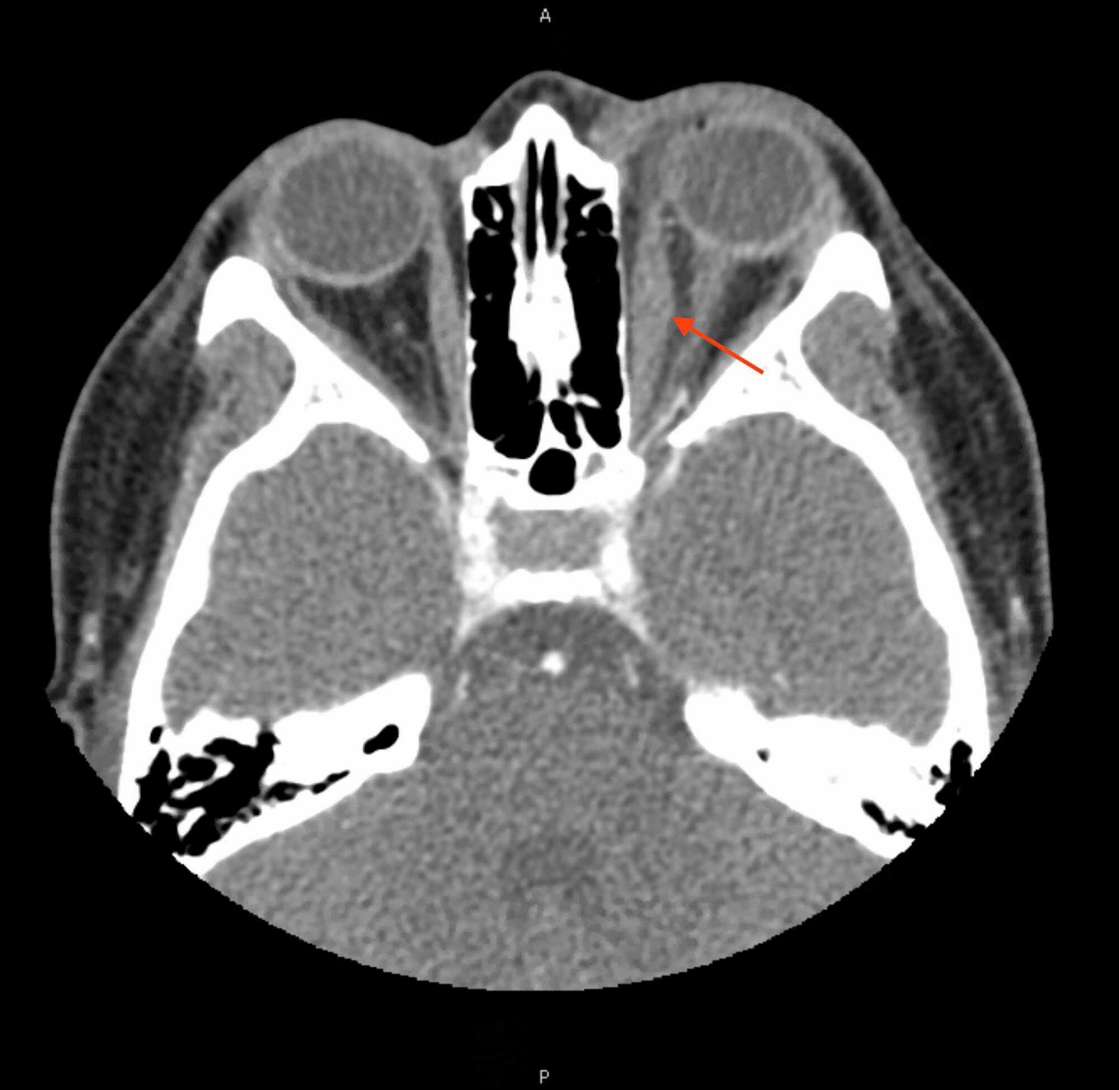
CT image with contrast showing left medial rectus swelling CT = computed tomography. Red arrow denotes the left medial rectus.

Initial laboratory investigation including complete blood cell count and basic metabolic panel were normal (Table 1). Computed tomographic (CT) images with contrast showed inflammation of the medial rectus muscle with evidence of post- and pre-septal edema (Figure 2). There was no evidence of abscess or sinus disease. Given the constellation of symptoms and degree of inflammation seen on the CT images, the patient was diagnosed with probable orbital cellulitis. She was started on intravenous vancomycin and ampicillin-sulbactam and admitted to the pediatric hospitalist service for further management.

**Table 1:** Laboratory evaluation on admission mm = millimeter. g = gram. dL = deciliter. mmol = millimole. L = liter.

Laboratory Test	Reference Ranges	Initial Evaluation
White Blood Cells (thousand/mm^3^)	(4.0-10.0)	9.0
Neutrophil (%)	(37-84)	69
Lymphocyte (%)	(8-49)	23
Eosinophil (%)	(0-7)	1
Hemoglobin (g/dL)	(12.0-18.0)	14.1
Platelets (thousand/mm^3^)	(140-440)	262
Sodium (mmol/L)	(134-146)	144
Potassium (mmol/L)	(3.5-5)	4.3
Chloride (mmol/L)	(98-106)	107
Bicarbonate (mmol/L)	(18-31)	24
BUN (mg/dL)	(6-20)	8
Creatinine (mg/dL)	(0.59-0.86)	0.77
Glucose (mg/dL)	(70-106)	83
Calcium (mg/dL)	(8-11)	9.9

The patient completed over 48 hours of intravenous antibiotics without improvement in eye pain, swelling, or erythema. At this time, magnetic resonance (MR) images showed nonspecific enhancement and enlargement of the left medial rectus muscle without abscess formation. Pediatric rheumatology and infectious disease specialists were consulted and both recommended discontinuing broad-spectrum intravenous antibiotics given the lack of both clinical improvement and progression of disease on diagnostic imaging. Additional lab testing was broadly normal (Table 2). After a multidisciplinary discussion with infectious diseases, rheumatology, and ophthalmology, a diagnosis of orbital myositis was considered, antibiotics were stopped, and an empiric trial of intravenous methylprednisolone (30mg/kg/dose) was administered. The patient showed dramatic clinical improvement within 24 hours of steroid administration with a significant reduction in her orbital pain, swelling, and erythema. She was subsequently discharged to complete an extended prednisone taper with rheumatology and ophthalmology follow-up. The final diagnosis was determined to be idiopathic orbital inflammation-orbital myositis variant.

**Table 2:** Laboratory evaluation on hospital day 3 mm = millimeter. g = gram. dL = deciliter. uL = microliter. ESR = erythrocyte sedimentation rate. mm = millimeter. hr = hour. CRP = C-reactive protein. mg = milligram. U = unit. L = Liter. uIU = micro International units. ng = nanogram.

Laboratory Test	Reference Ranges	Hospital Day 3
White Blood Cells (thousand/mm^3^)	(4.0-10.0)	10.7
Neutrophil (%)	(37-84)	67
Lymphocyte (%)	(8-49)	23
Eosinophil (%)	(0-7)	1
Hemoglobin (g/dL)	(12.0-18.0)	12.1
Platelets (thousand/mm^3^)	(140-440)	241
ESR (mm/hr)	(0-20)	27
CRP (mg/dL)	(0.01-0.3)	0.72
Albumin (g/dL)	(3.5-5.0)	3.1
Albumin/Globulin Ratio	(1.0-2.2)	1.0
Angiotensin Converting Enzyme (U/L)	(8-52)	28
Total CK (U/L)	(24-170)	69
Thyroid Stimulating Hormone (uIU/mL)	(0.3-4.2)	2.83
Free T-4 (ng/dL)	(0.8-1.8)	1.18
Thyroglobulin Antibody (IU/mL)	(<4.0)	<1.0
Thyroid Peroxidase Antibody (IU/mL)	(0-34)	<10.0
C3 (mg/dL)	(81-145)	143

## Discussion

Idiopathic orbital inflammation (IOI; formerly known as orbital pseudotumor) is a term used to describe the enlargement of any orbital structure from nonspecific inflammation of unclear etiology and is generally considered a diagnosis of exclusion. While IOI most commonly affects the lacrimal gland or the ocular soft tissues, the involvement of extraocular muscles, as seen in the orbital myositis (OM) variant, is considered a rarer presentation [1-3]. Other rare presentations include involvement of the optic nerve sheath, sclera, and uvea [2, 3]. As observed in our case, the medial rectus is the most commonly described extraocular muscle group involved in the OM variant of IOI [1]. IOI has been well-described in the adult population, but only 6-17% of IOI occurs in pediatric patients with less than 100 cases described in the literature [1]. Although no definitive etiology has been established for IOI, there have been reported associations with both rheumatologic disease and preceding infection, including upper respiratory infection, streptococcal pharyngitis, Lyme disease, and herpesvirus [4-8].

Common presenting symptoms of IOI, such as visual changes, orbital swelling, proptosis, and reduced or painful extraocular movements, share significant overlap with orbital cellulitis. As was observed in our case, Spindle et al. demonstrated that 50% of pediatric patients with IOI were initially misdiagnosed with orbital cellulitis [1]. If clinical findings are potentially concerning for orbital cellulitis, initial treatment with broad-spectrum empiric antibiotics is warranted for at least 48 hours. If the patient fails to improve as expected, a broader workup must be initiated to rule out other etiologies, as IOI remains a diagnosis of exclusion. Further workup should include consideration for possible abscess formation and/or worsening infection (MRI or CT with contrast), thyroid eye disease (thyroid-stimulating hormone, T4), sarcoidosis (angiotensin-converting enzyme, lysozyme, chest x-ray), Wegner’s (anti-neutrophil cytoplasmic antibodies), and neoplasm (complete blood count, MRI or CT). Other less likely etiologies to consider include Langerhans cell histiocytosis, ruptured dermoid cyst, and infectious dacryoadenitis. While there are recently proposed criteria for the diagnosis of exclusion for IOI in adults, no such criteria currently exist within the field of pediatrics [4].

In suspected IOI, the utility of a biopsy is controversial [5-7]. Generally, the lesion is biopsied unless it is solely myositic or perineural where the risk of iatrogenic damage outweighs the benefits of tissue diagnosis [8, 9]. This process is echoed in the aforementioned diagnosis guidelines for adults that recommend the final step as biopsy if the lesion is non-myositic or an empiric steroid trial if the lesion is myositic [4]. Once the diagnosis of IOI is confirmed either via biopsy or exclusion of other likely causes, the mainstay of therapy is corticosteroids with an extended steroid taper. The patient is expected to have a drastic response to high dose steroids within 24 hours, as was seen in this case, and can make a potential diagnosis of IOI more likely. This initial steroid dosing should be followed with an extended taper over six to eight weeks to reduce the risk of recurrence. 

The long-term rate of disease recurrence ranges from 37% to 76%, necessitating close outpatient follow-up with both ophthalmology and rheumatology [1, 10]. In a multicenter, international case series, investigators found that recurrence is more frequent in females or patients with bilateral IOI [1]. The pediatric literature is limited to case reports and there is a paucity of literature on recommendations for the management of recurrent pediatric IOI. In adult patients with recurrent IOI, treatment often consists of additional courses of corticosteroids, methotrexate, oral cyclosporine-A (CsA), rituximab, intravenous immunoglobulin, or radiation therapy [11]. More recently, there have been reports of using infliximab or topical corticosteroids in conjunction with topical CsA to manage refractory cases [12, 13].

There is a clear overlap with the clinical presentation of IOI and orbital cellulitis and differentiating the two disease processes can be difficult. Our patient did not have an elevated white blood cell count, fever, or evidence of sinus disease on admission, which made orbital cellulitis less likely. While there are reports of treating orbital cellulitis with concurrent antibiotics and corticosteroids [14], concurrent treatment would likely have obfuscated the diagnosis of IOI in our case. This would have resulted in her discharge with unnecessary antibiotics and without the appropriate steroid taper to maximize her likelihood of durable remission.

## Conclusions

This case highlights the importance of maintaining a high index of suspicion when the initial diagnosis does not progress as expected. Although orbital cellulitis is significantly more common than OM, considering a broad differential for periorbital swelling can help tailor the diagnostic and treatment approach.
